# Physical Fitness Level Is Related to Attention and Concentration in Adolescents

**DOI:** 10.3389/fpsyg.2020.00110

**Published:** 2020-02-04

**Authors:** Rafael E. Reigal, Luna Moral-Campillo, Rocío Juárez-Ruiz de Mier, Juan P. Morillo-Baro, Verónica Morales-Sánchez, José L. Pastrana, Antonio Hernández-Mendo

**Affiliations:** ^1^University of Málaga, Málaga, Spain; ^2^Department of Evolutionary Psychology and Education, University of Málaga, Málaga, Spain; ^3^Department of Social Psychology, Social Work, Anthropology and East Asian Studies, University of Málaga, Málaga, Spain; ^4^Department of Languages and Computer Science, University of Málaga, Málaga, Spain

**Keywords:** attention, concentration, adolescents, physical fitness, sports activity

## Abstract

The main goal of this study was to analyze the relationships among physical fitness, selective attention and concentration in a group of 210 teenagers (43.81% male, 56.19% female) from the city of Málaga (Spain), aged between 11 and 15 years old (*M* = 13.27, *SD* = 1.80) that participated in the study. D2 attention test was used in order to analyze selective attention and concentration. Physical fitness was evaluated using the horizontal jump test, the Course Navette test and the 5 × 10 meters speed test. The analysis taken indicated a significant relationship among the physical fitness level, the attention and the concentration, as in the general sample as looking at gender. Linear regression tests performed showed that oxygen consumption is the best predictor of attentional parameters. Cluster analysis shows two groups characterized by a greater or lower physical fitness level. So, the highest physical fitness level group scores better in the attention (e.g., boys: *p* < 0.001, *d*’ Cohen = 1.01, 95% CI [0.57, 1.44]; girls: *p* < 0.01, *d*’ Cohen = 0.61, 95% CI [0.24, 0.98]) and the concentration tests (e.g., boys: *p* < 0.001, *d*’ Cohen = 0.89, 95% CI [0.46, 1.32]; girls: *p* < 0.01, *d*’ Cohen = 0.58, 95% CI [0.21, 0.95]). Results indicate that physical fitness analysis can be used as a tool for observing differences in the attention and concentration level of the analyzed adolescents, suggesting that a physical performance improvement could be an adequate procedure to develop some cognitive functions during adolescence.

## Introduction

Brain functioning study has increased in the field of physical activity and sports sciences in recent years (Northey et al., 2018; Xue et al., 2018; Reigal et al., 2019b). There is a large number of studies analyzing the practice of physical activity and cognitive functioning in children and adolescents showing links between these variables (Donnelly et al., 2016; Chaddock et al., 2018). For example, positive relationships have been observed at early ages between regular or acute physical activity with cognitive abilities such as attention, concentration, memory, working memory, cognitive flexibility, inhibitory control or processing speed (Best, 2010; Tomporowski et al., 2011; Scudder et al., 2014; Li et al., 2017). These findings are especially relevant in stages such as childhood or adolescence, because an appropriate cognitive development would help to a better psychosocial adjustment and adaptation to the environment, as well as good mental health and a higher quality of life (Costigan et al., 2016; Santana et al., 2017; Zmyj et al., 2017).

However, while physical activity is considered to be positively related to cognitive functioning, researching has shown that it is necessary to assess the level of physical fitness for a better understanding of these links (Hillman et al., 2009; Herting et al., 2014; Pérez-Lobato et al., 2016). Physical fitness would refer to a set of physical characteristics that people have or could achieve through an adaptation process. In general, good physical fitness allows physical exertion without fatigue and vigorousness. Among others, some of the factors that are often measured for evaluation are cardiorespiratory fitness, muscle strength or body composition (Caspersen et al., 1985). Authors such as Hillman et al. (2009) have shown that physical condition is an essential element to explain the relationships between physical activity and cognitive functioning. Previous research has shown that brain development in childhood and adolescence is conditioned by factors linked to health, and the level of physical condition can be an indicator of brain health (Hötting and Röder, 2013; Reloba-Martínez et al., 2017).

What it means that physical activity taken should be done with a certain level of exigency in order to have a significant impact on the participants. Physical fitness development is a sign of the impact that physical activity has on human body, which it also corresponds with better effects in the brain. Working on that researching line, authors such as Pontifex et al. (2011), Kao et al. (2017), Reloba-Martínez et al. (2017) or Westfall et al. (2018) have pointed out positive associations between physical fitness and cognitive functioning in children and adolescents, with aerobic capacity being the best predictor of these relationships. However, other manifestations of physical condition such as motor control or strength are being explored in recent years, and it has been observed that they can have a positive influence on brain development (Esteban-Cornejo et al., 2019).

In the set of cognitive functions, attention has been one of the most attentions received in adolescence stage (Kim-Spoon et al., 2015; Bauer and Manning, 2016). This is a basic cognitive capacity involved in a broad set of tasks, conditioning the probability of success of behavior in areas such as academic, sports or social (Perlman et al., 2014; Rabiner et al., 2016). In addition, attention is not a univocal construction, but presents different manifestations as arousal, focal, selective, divided, alternating or sustained (Manly et al., 2001; Tamm et al., 2013). Selective attention refers to the ability to attend to objective stimuli while ignoring other distracting stimuli (Estévez-González et al., 1997; Giuliano et al., 2014). Likewise, the ability to maintain attention accurately and to a high degree on a given stimulus is called concentration (Buehner et al., 2006). Performing a task with high levels of concentration and for a long time is called sustained attention, which is necessary to succeed in a broad set of tasks (Blotenberg and Schmidt-Atzert, 2019).

Several studies have shown that physical activity in childhood and adolescence is positively related to improved attention and concentration (Budde et al., 2008; Trudeau and Shephard, 2008; Verret et al., 2012; Vanhelst et al., 2016). Specifically, previous studies have indicated the positive effects of physical activity on selective attention in children and adolescents (Janssen et al., 2014; Tine, 2014; Altenburg et al., 2016). Other works have related physical fitness level in children and adolescents with selective attention and concentration, observing that cardiorespiratory aptitude is one of the variables that explains better these associations (Pérez-Lobato et al., 2016; Reloba-Martínez et al., 2017; Reigal et al., 2019a).

Although there are previous studies that have explored this phenomenon, some of them have evaluated only isolated physical capacities, others have not taken a differentiated gender analysis, and a few have grouped different manifestations of physical fitness into profiles. In addition, we would like to evaluate the predictive capacity of fitness on cognitive functioning. For all this, aiming to go in depth into the relationships between physical fitness, selective attention and concentration, this paper focus on: (1) Analyzing the correlations among these variables according to gender; (2) assess the predictive capacity of fitness on selective attention and concentration; (3) measuring differences in selective attention and concentration based on fitness and gender.

## Materials and Methods

### Participants

A total of 210 adolescents (43.81% male, 56.19% female) from the city of Málaga (Spain), aged between 11 and 15 years old (*M* = 13.27, *SD* = 1.80) participated in this research. Sample was selected through a suitable sampling group from several schools in Malaga. The exclusion criteria were: (a) not between 11 and 15 years old, (b) have a health problem that could bias any result or prevent you from taking a test of the study, and (c) not have parental authorization.

### Measures and Instruments

#### Attention Test d2 (Brickenkamp, 2002)

This test is used to analyze the ability to visually scan for target items quickly and accurately, ignoring irrelevant ones, which is considered a manifestation of selective attention and concentration. In this test it is necessary to discriminate among 47 characters in 14 rows, with a total of 658 elements. You have 20 s to complete each row. Stimuli contain the letters “d” or “p,” which may be accompanied by one or two lines on the top of the item, on the bottom, or both. The “d” must be crossed out with two stripes (regardless of position). The test is always completed from left to right and from top to bottom. The scores you get are: TR (processed elements), TA (successes), O (omissions), C (commissions or errors), TOT [effectiveness in the task = TR−(O + C)], CON (concentration = TA−C), TR+ (last stimulus analyzed in the row with the most attempted elements), TR− (last stimulus analyzed in the row with the least attempted elements) and VAR [index of variation between the last stimulus analyzed between different rows = (TR+)−(TR−)]. This test has a test–retest reliability in the original study up to 0.90.

#### Physical Fitness

Three tests, which are included in the Eurofit (1993), were used to analyze the physical fitness of the participants: the horizontal Jump test (to evaluate the explosive strength in the lower limbs), the 5 × 10 meters speed test (to analyze the speed and the agility) and the Course Navette test (Léger et al., 1988) (to assess cardiorespiratory fitness). The latter test allows to calculate indirectly the maximum oxygen consumption (VO_2max_). To calculate the maximum oxygen consumption the formula VO_2max_ = 31.025+3.238S−3.248A+0.1536SA was used (*S* = speed reached in the last completed stage; *A* = age).

### Procedure

Sample set was obtained from schools. Every school management was contacted in order to request them to participate. In addition, written and informed consent was obtained from the parent/guardian of each adolescent who wanted to participate. Authorization was also obtained from the Ethics Committee of the University of Malaga (CEUMA, n° 243, 18-2015-H) and the principles of the Declaration of Helsinki were complied with throughout the phases of the study (World Medical Association [WHO], 2013). Evaluations were taken along 2 days with a 3 days difference between them. Attention and concentration were evaluated in the first day, and physical fitness in the second day. The evaluation schedules were from 9:00 to 12:00 in the morning.

The D2 test was taken following the protocol established in the test. Instructions were carefully explained to the students and previous questions were solved in order to avoid any doubts. The test was conducted in groups in a classroom at the school.

For the physical tests, a 15-min warm-up was carried out. Activation, joint mobility and explosive exercises were developed, as well as specific tests of the horizontal jump and speed tests. Subsequently, the tests were taken, with a 15-min break between them and following the same order: horizontal jump test, 5 × 10-meter speed test and Course Navette test, following the protocols established by the Eurofit (1993).

### Data Analysis

Descriptive and inferential analysis were performed. Data normality was checked with the Kolmogorov–Smirnov test. Correlation analyses were performed with the Pearson and Spearman test (±0.01 to ±0.19 = very weak correlation; ±0.20 to ±0.39 = weak correlation; ±0.40 to ±0.59 = moderate correlation). The predictive capacity of fitness on selective attention and concentration was assessed by linear regression analysis (successive steps). Different clusters were established according to fitness level and gender by k-mean clustering analysis. Also, comparisons between groups were made with *t*-student and *U*-Mann Whitney tests. The IBM SPSS Statistics 24.0 statistical analysis package was used to process the data.

## Results

Table 1 shows the descriptive statistics and normality test (Kolmogorov–Smirnov) of the variables under study. Only the variable commissions (C) of test d2 showed problems of normality.

**TABLE 1:** Descriptive statistics of study variables and Kolmogorov–Smirnov test.

	**Total (*n* = 210)**					**Boys (*n* = 92)**					**Girls (*n* = 118)**				
	***M***	***SD***	***S***	***K***	***K–S***	***M***	***SD***	***S***	***K***	***K–S***	***M***	***SD***	***S***	***K***	***K–S***
HJT	158.06	33.08	0.44	–0.34	1.08	176.16	35.06	–0.11	–0.86	0.85	143.94	23.23	0.04	–0.24	1.11
5 × 10	19.17	1.97	0.61	0.16	1.16	18.23	1.77	0.98	0.99	0.97	19.90	1.82	0.69	0.26	1.22
VO_2max_	43.05	7.72	0.34	0.98	1.10	45.52	8.96	0.11	0.57	0.98	41.13	5.97	–0.19	0.02	0.92
D2-TR	59.26	19.56	0.08	–0.70	1.03	56.02	20.40	0.31	–0.49	0.91	61.79	18.58	–0.07	–0.75	1.17
D2-TA	58.90	19.74	0.07	–0.73	1.08	57.38	21.01	0.21	–0.78	0.76	60.09	18.69	–0.04	–0.65	0.81
D2-O	53.18	21.05	0.00	–0.37	0.98	56.22	22.67	–0.16	–0.75	0.85	49.55	19.00	–0.03	0.16	0.86
D2-C	52.65	17.06	–0.40	–0.45	2.83a	53.62	17.51	–0.17	–0.50	2.04a	51.90	16.74	–0.61	–0.46	1.96a
D2-TOT	60.14	19.23	0.02	–0.76	1.27	57.85	20.49	0.23	–0.64	0.87	61.93	18.08	–0.12	–0.83	1.12
D2-CON	59.28	19.65	0.14	–0.81	1.20	57.95	21.45	0.23	–0.91	0.94	60.31	18.15	0.10	–0.74	0.88
D2-VAR	52.40	21.06	–0.21	–0.52	1.04	50.92	21.08	–0.20	–0.67	0.65	52.19	21.13	–0.22	–0.37	0.90
D2-TR+	60.94	16.68	0.01	–0.24	1.08	59.32	17.82	0.02	–0.43	0.97	62.20	15.69	0.08	–0.08	0.98
D2-TR−	62.68	20.05	0.31	–0.80	1.23	61.87	20.62	0.48	–0.99	1.27	63.31	19.65	0.17	–0.55	1.21

**M* = Mean; *SD* = standard deviation; *S* = Skewness; *K* = Kurtosis; *K–S* = Kolmogorov–Smirnov test; HJT = Horizontal jump (cm); 5 × 10 = Speed test 5 × 10 meters (sec.); VO_2max_ = Maximum oxygen consumption (ml/kg/min); D2 = d2 test; TR = Total number of attempts; TA = Total successes; O = Omissions; C = Commissions; TOT = Total effectiveness in the test; CON = Concentration index; VAR = Variation index; TR+ = Line with the greatest number of elements attempted; TR− = Line with the least number of elements attempted. ^*a*^*p* < 0.001.*

Correlation analyses (Table 2) indicated significant and positive relationships between the variables horizontal jump and maximum oxygen consumption with various measures of the D2 attention test. Also, the speed test was significantly and negatively associated with some of the attention test measures. However, the omissions, commissions and index of variation (D2 test) were not related to any of the physical fitness variables. In the general sample, the correlations among horizontal jump and speed test with the main measures of D2 test are weak. However, the correlation level between maximum oxygen consumption with TOT and CON are moderate (*r* ≥ 0.40). On the other hand, correlations in boys are greater than in girls. Children have moderate or near-moderate correlations between the main measures of the D2 test and physical fitness variables (horizontal jump and maximum oxygen consumption, *r* = 0.49–0.54; speed test, *r* ≤ −0.36 to −0.37). However, in girls the correlations are weaker (horizontal jump and maximum oxygen consumption, *r* = 0.28–0.38; speed test, *r* = −0.16 to −0.20).

**TABLE 2:** Correlation analysis among D2 test and physical fitness factors by gender and total sample.

	**Total**			**Boys**			**Girls**		
	**HJT**	**5 × 10**	**VO_2max_**	**SH**	**5 × 10**	**VO_2max_**	**SH**	**5 × 10**	**VO_2max_**
D2-TR	0.30***	−0.20**	0.39***	0.52***	−0.37***	0.54***	0.33***	−0.23*	0.35***
D2-TA	0.33***	−0.20**	0.40***	0.50***	−0.36**	0.49***	0.32***	–0.16	0.39***
D2-O	–0.04	0.01	–0.02	–0.14	0.01	–0.15	–0.14	0.14	0.05
D2-C	–0.10	0.08	0.04	–0.16	0.14	–0.03	–0.12	0.10	0.09
D2-TOT	0.32***	−0.21**	0.41***	0.51***	−0.37**	0.54***	0.32**	−0.20*	0.36***
D2-CON	0.33***	−0.20**	0.40***	0.50***	−0.36**	0.49***	0.28**	–0.16	0.38***
D2-VAR	–0.07	0.06	0.08	–0.18	0.07	0.08	0.11	0.03	0.10
D2-TR+	0.04	0.02	0.29***	0.04	0.05	0.34***	0.17	–0.07	0.33***
D2-TR−	0.32***	−0.19**	0.24***	0.54***	−0.32**	0.37***	0.21*	–0.16	0.15

HJT = Horizontal jump (cm); 5 × 10 = Speed test 5 × 10 meters (sec.); VO_2max_ = Maximum oxygen consumption (ml/kg/min); D2 = d2 test; TR = Total number of attempts; TA = Total number of hits; O = Omissions; C = Commissions; TOT = Total effectiveness in the test; CON = Concentration index; VAR = Variation index; TR+ = Line with the greatest number of attempted elements; TR− = Line with the least number of attempted elements. * *p* < 0.05; ** *p* < 0.01; *** *p* < 0.001.

The linear regression analysis (successive steps) can be seen in Table 3. The latest models generated for the whole sample and differentiating by gender are shown. Some variables were excluded as predictors due to the lack of significance (*p* > 0.05). The linearity assumptions were met in the relationship between predictor variables and criteria, homoscedasticity and normal waste distribution. The value of Durbin–Watson was between 1.69 and 2.34, so it can be assumed that the waste is independent and the assumption of independence of the independent variables with respect to the dependent one is fulfilled (Pardo and Ruiz, 2005). Tolerance Index (ranged from 0.66 to 0.99) and Variance Inflation Factor (VIF) (ranged from 1.01 to 1.52) did not reveal issues of multicollinearity.

**TABLE 3:** Linear regression analysis of D2 test factors regressed on physical fitness predictors by gender and total sample.

		**Total sample**			**Boys**			**Girls**		
		***B***	***SE B***	**β**	***B***	***SE B***	**β**	***B***	***SE B***	**β**
D2-TR	VO_2max_	1.04	0.15	0.43***	0.85	0.23	0.37***	0.76	0.32	0.24*
	5 × 10	–	–	–	–	–	–	–	–	–
	HJT	–	–	–	0.19	0.06	0.32**	0.16	0.08	0.20*
		(*R*^2^ = 0.19; *F* = 46.95***)			(*R*^2^ = 0.35; *F* = 25.88***)			(*R*^2^ = 0.14; *F* = 10.43***)		
D2-TA	VO_2max_	0.84	0.19	0.35***	0.73	0.24	0.31**	1.22	0.27	0.39***
	5 × 10	–	–	–	–	–	–	–	–	–
	HJT	0.09	0.04	0.17*	0.20	0.06	0.34**	–	–	–
		(*R*^2^ = 0.22; *F* = 28.34***)			(*R*^2^ = 0.31; *F* = 21.28***)			(*R*^2^ = 0.14; *F* = 20.61***)		
D2-TOT	VO_2max_	1.01	0.15	0.42***	0.84	0.23	0.37***	1.10	0.26	0.36***
	5 × 10	–	–	–	–	–	–	–	–	–
	HJT	–	–	–	0.19	0.06	0.32**	–	–	–
		(*R*^2^ = 0.18; *F* = 44.59***)			(*R*^2^ = 0.35; *F* = 25.09***)			(*R*^2^ = 0.13; *F* = 17.74***)		
D2-CON	VO_2max_	1.09	0.16	0.45***	0.75	0.25	0.31**	1.16	0.26	0.38***
	5 × 10	–	–	–	–	–	–	–	–	–
	HJT	–	–	–	0.20	0.06	0.33**	–	–	–
		(*R*^2^ = 0.19; *F* = 50.11***)			(*R*^2^ = 0.31; *F* = 21.11***)			(*R*^2^ = 0.14; *F* = 19.52***)		
D2-TR+	VO_2max_	0.58	0.13	0.31***	0.68	0.20	0.34***	0.86	0.23	0.33***
	5 × 10	–	–	–	–	–	–	–	–	–
	HJT	–	–	–	–	–	–	–	–	–
		(*R*^2^ = 0.09; *F* = 21.41***)			(*R*^2^ = 0.11; *F* = 11.90***)			(*R*^2^ = 0.10; *F* = 13.90***)		
D2-TR−	VO_2max_	–	–	–	–	−	–	–	–	–
	5 × 10	–	–	–	–	–	–	–	–	–
	HJT	0.25	0.04	0.42***	0.36	0.05	0.64***	0.18	0.08	0.21*
		(*R*^2^ = 0.17; *F* = 43.47***)			(*R^2^* = 0.39; *F* = 57.93***)			(*R*^2^ = 0.04; *F* = 5.46*)		

*B* = Unstandardized beta coefficient; SE *B* = Standard error on beta; β = Standardized coefficient Beta; VO_2max_ = Maximum oxygen consumption; 5 × 10 = Speed test 5 × 10 meters; HJT = Horizontal jump test; D2 = d2 test; TR = Total Number of Attempts; TA = Total Hit; TOT = Total Test Effectiveness; CON = Concentration Index; TR+ = Line with the greatest number of attempted elements; TR− = Line with the least number of attempted elements. * *p* < 0.05; ** *p* < 0.01; *** *p* < 0.001.

**FIGURE 1:**
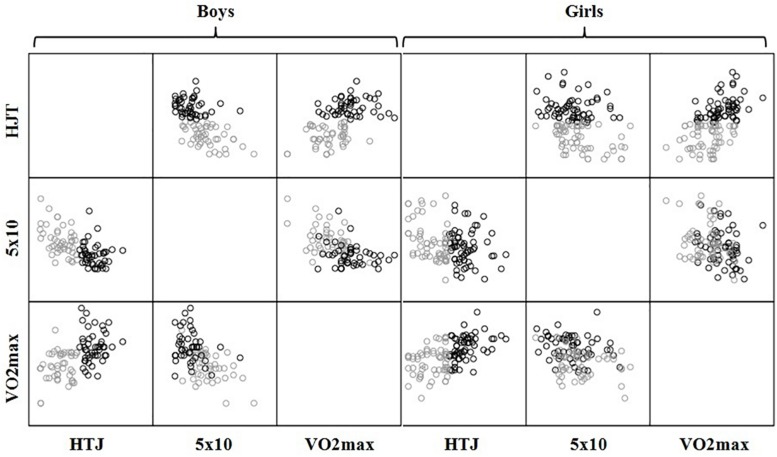
Clusters generated from physical fitness variables. HJT = Horizontal jump test; 5 × 10 = Speed test 5 × 10 meters; VO_2max_ = Maximum oxygen consumption.

K-means clustering was used to generate two groups. The groups constituted were characterized by lower level of physical fitness (group 1) (boys, *n* = 45; girls, *n* = 57) and higher level of physical fitness (group 2) (boys, *n* = 47; girls, *n* = 61) (Figure 1). The clusters were generated depending on the following variables: horizontal jump test, speed test 5 × 10 and VO_2max_. Each data was correctly classified, because the maximum distance of each one from the center of its group was less than the distance between the centers of the clusters.

Tables 4, 5 show descriptive and normal measures (Kolmogorov–Smirnov) of the different clusters for each gender. In addition, Table 4 shows the significant differences calculated with the *t-student* statistic. Differences for the commissions (C) measure of the D2 attention test were calculated with the *U*-Mann Whitney test but they were not significant. As can be seen, there were differences for boys between clusters in the measures TR (*p* < 0.001, *d*’ Cohen = 1.05, 95% CI [0.62, 1.49]), TA (*p* < 0.001, *d*’ Cohen = 0.90, 95% CI [0.47, 1.33]), TOT (*p* < 0.001, *d*’ Cohen = 1.01, 95% CI [0.57, 1.44]), CON (*p* < 0.001, *d*’ Cohen = 0.89, 95% CI [0.46, 1.32]) and TR− (*p* < 0.001, *d*’ Cohen = 1.16, 95% CI [0.72, 1.60]) of the D2 attention test, as well as in the horizontal jump test (*p* < 0.001, *d*’ Cohen = 3.29, 95% CI [2.67, 3.93]), 5 × 10 speed test (*p* < 0.001, *d*’ Cohen = −1.15, 95% CI [−1.59, −0.71]) and VO_2max_ (*p* < 0.001, *d*’ Cohen = 1.27, 95% CI [0.82, 1.72]). Also, there were differences for girls between clusters in the measures TR (*p* < 0.01, *d*’ Cohen = 0.58, 95% CI [0.21, 0.95]), TA (*p* < 0.01, *d*’ Cohen = 0.61, 95% CI [0.24, 0.98]), TOT (*p* < 0.01, *d*’ Cohen = 0.61, 95% CI [0.24, 0.98]), CON (*p* < 0.01, *d*’ Cohen = 0.58, 95% CI [0.21, 0.95]) and TR− (*p* < 0.05, *d*’ Cohen = 0.42, 95% CI [0.05, 0.78]) of the D2 attention test, as well as in the horizontal jump test (*p* < 0.001, *d*’ Cohen = 2.59, 95% CI [2.10, 3.08]) and VO_2max_ (*p* < 0.001, *d*’ Cohen = 1.06, 95% CI [0.68, 1.45]).

**TABLE 4:** Descriptive statistics of study variables analyzed by cluster and differences among groups (*t*-student).

	**Boys**					**Girls**				
	**Low PF (45)**		**High PF (47)**			**Low PF (57)**		**High PF (61)**		
	***M***	***SD***	***M***	***SD***	***t***	***M***	***SD***	***M***	***SD***	***t***
HJT	145.60	19.33	205.43	16.91	15.82***	124.93	14.36	161.70	14.00	14.08***
5 × 10	19.14	1.72	17.37	1.34	−5.34***	20.22	1.91	19.60	1.69	–1.86
VO_2max_	40.61	7.37	50.22	7.78	6.08***	38.22	5.58	43.85	4.99	5.78***
D2-TR	46.29	17.45	65.34	18.73	5.04***	56.44	18.19	66.79	17.66	3.14**
D2-TA	48.51	18.07	65.87	20.24	4.33***	54.40	17.93	65.41	17.94	3.33**
D2-O	59.33	21.26	53.23	23.78	–1.30	50.65	18.28	48.52	19.74	–0.61
D2-C	56.64	16.69	50.72	17.97	–1.64	52.05	16.35	51.75	17.23	–0.09
D2-TOT	48.40	17.69	66.89	18.98	4.83***	56.49	16.98	67.02	17.71	3.29**
D2-CON	48.98	18.17	66.53	20.99	4.28***	55.07	17.37	65.21	17.62	3.15**
D2-VAR	54.29	19.80	47.70	20.71	–1.55	52.81	21.33	51.62	21.09	–0.30
D2-TR+	58.93	19.69	59.68	16.04	0.20	60.82	15.47	63.49	15.92	0.92
D2-TR−	51.27	15.56	72.02	19.87	5.56***	59.14	20.21	67.20	18.44	2.27*

HJT = Horizontal jump (cm); 5 × 10 = Speed test 5 × 10 meters (sec.); VO_2max_ = Maximum oxygen consumption (ml/kg/min); PF = Physical fitness; D2 = d2 test; TR = Total number of attempts; TA = Total successes; O = Omissions; C = Commissions; TOT = Total effectiveness in the test; CON = Concentration index; VAR = Variation index; TR+ = Line with the greatest number of attempted elements; TR− = Line with the least number of attempted elements. * *p* < 0.05 ** *p* < 0.01 *** *p* < 0.001.

**TABLE 5:** Skewness, Kurtosis and Kolmogorov–Smirnov by cluster and gender.

	**Boys**						**Girls**					
	**Low PF**			**High PF**			**Low PF**			**High PF**		
	***S***	***K***	***K–S***	***S***	***K***	***K–S***	***S***	***K***	***K–S***	***S***	***K***	***K–S***
HJT	–0.34	–0.87	0.86	0.72	0.30	0.94	–0.34	–1.31	1.27	1.23	1.24	1.08
5 × 10	0.79	0.70	0.67	1.64	0.94	0.92	0.84	0.10	1.07	0.42	–0.01	0.71
VO_2max_	–0.40	0.52	1.08	0.49	0.17	0.94	–0.19	–0.25	0.92	0.04	0.09	0.77
D2-TR	0.68	0.58	0.86	0.10	–0.56	0.64	0.41	–0.29	1.32	–0.52	–0.31	1.18
D2-TA	0.52	–0.02	0.75	–0.14	–0.80	0.53	0.26	–0.06	0.93	–0.33	–0.64	0.91
D2-O	–0.55	–0.25	0.88	0.38	–0.76	0.69	–0.33	0.99	0.79	0.21	–0.23	0.74
D2-C	–0.99	0.01	1.64*	0.50	0.04	1.23	–0.56	–0.66	1.28	–0.66	–0.27	1.47*
D2-TOT	0.56	0.41	1.02	–0.04	–0.83	0.75	0.16	–0.44	0.85	–0.45	–0.67	1.20
D2-CON	0.59	–0.01	0.97	–0.19	–0.95	0.71	0.43	0.01	0.86	–0.20	–0.87	0.96
D2-VAR	–0.22	–0.57	0.73	–0.10	–0.93	0.65	–0.27	–0.38	0.77	–0.18	–0.28	0.99
D2-TR+	0.15	–0.67	1.19	–0.17	–0.08	0.69	0.28	–0.05	1.15	–0.11	0.11	0.79
D2-TR−	1.45	2.10	1.23	–0.19	–1.11	0.93	0.55	–0.42	1.04	–0.13	–0.07	0.99

*S* = Skewness; *K* = Kurtosis; *K–S* = Kolmogorov–Smirnov; HJT = Horizontal jump test; 5 × 10 = Speed test 5 × 10 meters; VO_2max_ = Maximum oxygen consumption; PF = Physical fitness; D2 = d2 test; TR = Total number of attempts; TA = Total number of hits; O = Omissions; C = Commissions; TOT = Total effectiveness in the test; CON = Concentration index; VAR = Variation index; TR+ = Line with the greatest number of attempted elements; TR− = Line with the least number of attempted elements. * *p* < 0.05.

## Discussion

The purpose of present investigation was triple. On the one hand, it was to analyze the relations between physical condition, selective attention and concentration in a sample of adolescents according to gender. On the other hand, determine the predictive capacity of fitness on selective attention and concentration. Finally, it was intended to generate two groups based on the level of fitness and gender to assess differences in cognitive functioning measures. For the first and second objectives, correlation and linear regression tests have been carried out, observing that there were significant relationships in total sample as well as for each gender in the analyzed variables. It has also been observed that maximum oxygen consumption has been the main predictor of attention and concentration measures, although in boys strength is also a significant predictor. For the third objective, participants were classified into groups characterized by a higher and lower level of physical fitness and gender, getting better scores in selective attention and concentration those groups with a better level of physical fitness. Looking at data together, these data are in the same line with previous studies that had linked the level of physical fitness with improved cognitive functioning at these ages (Pontifex et al., 2011; Kao et al., 2017; Westfall et al., 2018) and specifically, those which had observed this phenomenon in selective attention and concentration (Trudeau and Shephard, 2008; Pérez-Lobato et al., 2016; Reloba-Martínez et al., 2017).

Correlation and linear regression analyses have highlighted that maximum oxygen consumption is an essential variable to explain the relationship between physical confidence and cognitive functioning. This is consistent with others studies that had observed this previously (Chaddock et al., 2010, 2016; Kao et al., 2017; Westfall et al., 2018). However, the explosive strength, which has been evaluated by the horizontal jump test, has also obtained adequate levels of correlation with selective attention measures and concentration. Although the relationship between aerobic capacity and cognitive functioning has been better established in previous research, the complexity of this phenomenon is so broad that more data on the underlying processes that accompany the improvement of other physical capacities and the mechanisms that allow it to be related it to brain development will probably be known in the future.

In fact, there are studies that have already highlighted the relationships between aspects such as strength or coordination skills and cognitive functioning in children and adolescents (Haapala et al., 2015; Mierau et al., 2016). For example, Esteban-Cornejo et al. (2017) consider that it is necessary to assess how the interaction of different physical qualities could modulate the brain development. Landrigan et al. (2019) performed a meta-analysis and observed the effects of strength training on cognitive functioning, highlighting the importance in the development of this physical quality. These authors consider that both the cognitive demands of the exercise itself and other physiological effects derived from physical exertion would explain this phenomenon. For example, some current hypotheses consider that physical exercise promotes the activity of biomolecules such as the brain-derived neurotrophic factor (BDNF) or the insulin-like growth factor-1 (IGF-1), which promote changes in brain (Tari et al., 2019).

Thus, the structural and functional changes in the brain were previously caused by improvements in fitness levels. In previous studies such as Esteban-Cornejo et al. (2017), it was observed that several factors of physical fitness, and similar to those analyzed in this research (oxygen consumption, speed-agility and strength), were related to the gray matter volume of some brain regions. This could be one of the reasons for the relationship between the physical condition and the cognitive functioning. By affecting the structural development of the brain, it would be conditioning its functioning. Specifically, these authors observed that cardiorespiratory fitness was best associated with the gray matter volume of cortical and subcortical brain areas. In this work, although the other measures of physical condition are also related to the levels of attention and concentration, it is observed that oxygen consumption is the variable that best explains the results in general terms. There are also other reasons that would explain the results. For example, physical training generates cognitive demands that have an impact on the brain. In addition, physiological processes are implemented as increased levels of neurotrophic or hormonal factors that facilitate brain plasticity processes (Landrigan et al., 2019).

These analyses have shown that there are differences among boys and girls in the level of correlation and in the predictive capacity of the variables. Data indicate that boys have better levels of correlation between the variables and the percentage of explained variance in the regression models are higher, which is consistent with previous studies (Drollette et al., 2015; Jaakkola et al., 2015). In addition, in boys strength is a more decisive factor. These differences could be conditioned by a higher level of regular physical activity in boys, which could lead to greater intra-group variability (Telford et al., 2016). In addition, other elements such as differences in brain structure, plasticity or the impact of environmental factors among genders may be influencing these results (Cosgrove et al., 2007; Isaacs et al., 2008). However, these are hypothesis that cannot be tested, but it would be interesting to integrate it into future work to check if gender could be subtly modulating the results.

On the other hand, this work has analyzed intragender differences based on the level of physical condition. This is interesting, as previously discussed, since it allows to observe whether this phenomenon reproduces similarly in both genres. In many previous studies, no analysis has been carried out on boys and girls to facilitate their comparison, which is an interesting aspect of this work. Based on the results obtained in this study, it can be observed that the size of the effect on intra-group differences in boys is greater than girls, although in both girls and boys the group with higher kevel f physical fitness had better scores in attentional and concentration measures. This raises the question of whether other variables such as study habits, rest and eating could also contribute to qualify the results found (Cosgrove et al., 2007; Isaacs et al., 2008). These suggestions should be addressed in future work, which will help to set a better description of the relationships between the variables analyzed. In any case, and despite the differences, these data are relevant because it would suggest that this phenomenon is fairly stable and can benefit a wide range of adolescents.

This research presents limitations such as the type of design, which does not allow to set causal relationships. In addition, assessments of other measures of physical fitness or cognitive functioning may provide more information about the findings. However, it provides an adequate sample size and gender analysis, which allows for selective in-depth analysis of boys and girls. Therefore, it is considered that the results found contribute as valuable information to the existing literature set that contributes to consolidate these relationships and increase the available evidence on this subject.

Results highlight that physical fitness in children and adolescents could help to improve their cognitive functioning, with the benefits this would bring to their developmental and psychosocial adjustment processes (Hillman et al., 2009; Pontifex et al., 2011; Herting et al., 2014; Kao et al., 2017). These data joined to previous ones, allow us to emphasize that the practice of physical activity alone is not enough for the impact on the brain to be noticeable. Physical activity must have specific qualities that allow it to overcome a minimum threshold of adaptation that facilitates changes in the organism (Chaddock et al., 2010; Esteban-Cornejo et al., 2017; Reloba-Martínez et al., 2017).

## Data Availability Statement

The datasets generated for this study are available on request to the corresponding author.

## Author Contributions

AH-M, VM-S, JP, RR, JM-B, RJ-R, and LM-C participated in the study design and data collection, performed the statistical analyses, contributed to the interpretation of the results, wrote the manuscript, approved the final manuscript as presented, reviewed and provided feedback to the manuscript, and made substantial contributions to the final manuscript. RR, AH-M, and LM-C conceived the study and participated in its design and coordination.

## Conflict of Interest

The authors declare that the research was conducted in the absence of any commercial or financial relationships that could be construed as a potential conflict of interest.
